# Clinical Significance of Peripheral Blood T Lymphocyte Subsets in *Helicobacter pylori*-Infected Patients

**DOI:** 10.1155/2012/819842

**Published:** 2012-03-28

**Authors:** Yuka Satoh, Hatsue Ogawara, Osamu Kawamura, Motoyasu Kusano, Hirokazu Murakami

**Affiliations:** ^1^Department of Laboratory Sciences, Course of Health Sciences, Gunma Graduate School of Health Sciences, Showa-machi 3-39-22, Maebasi 371-8514, Gunma, Japan; ^2^Department of Medicine and Molecular Science, Gunma University Graduate School of Medicine, Showa-machi 3-39-22, Maebasi 371-8511, Gunma, Japan; ^3^Department of Endoscopy and Endoscopic Surgery, Gunma University Hospital Showa-machi 3-39-15, Maebasi 371-8511, Gunma, Japan

## Abstract

*Background*. *Helicobacter pylori* chronically colonizes gastric/duodenal mucosa and induces gastroduodenal disease and vigorous humoral and cellular immune responses. *Methods*. In order to clarify the immunological changes induced by this infection, we determined the percentage and, as indicated, ratios of the following cells in peripheral blood of 45 *H. pylori*-infected patients and 21 control subjects: CD4^+^ T cell, CD8^+^ T cells, T helper 1 cells (Th1), T helper 2 cells (Th2), CD4^+^CD25^+^ T cells, Foxp3^+^ regulatory T cells (Tregs), CD4/CD8 ratio, and Th1/Th2 ratio. *Results*. The percentage of CD8^+^ T cells was significantly lower in *H. pylori*-infected patients (mean ± SD; 18.0 ± 7.1%) compared to control subjects (mean ± SD; 23.2 ± 7.8%) (*P* < 0.05). The CD4/CD8 ratio was significantly higher in *H. pylori*-infected patients (mean ± SD; 3.1 ± 2.4) compared to control subjects (mean ± SD; 2.1 ± 1.0) (*P* < 0.05). The Th1/Th2 ratio was significantly lower in *H. pylori*-infected patients (mean ± SD; 10.0 ± 8.5) compared to control subjects (mean ± SD; 14.5 ± 9.0) (*P* < 0.05). The percentage of CD4^+^CD25^+^ T cells in *H. pylori*-infected patients (mean ± SD; 13.2 ± 6.2%) was significantly higher than that in control subjects (mean ± SD; 9.8 ± 3.4%) (*P* < 0.05). However, there was no significant difference in Tregs. *Conclusion*. Tregs did not decrease, but the activation of humoral immunity and Th2 polarization were observed in the peripheral blood of *H. pylori*-infected patients. In some cases, these changes may induce systemic autoimmune diseases.

## 1. Introduction

*Helicobacter pylori* is a gram-negative, highly motile, and spiral-shaped bacterium, which is 2.5–3.5 *μ*m long and 0.5–1.0 *μ*m in diameter and has one to six polar flagella at one end. *H. pylori* was discovered in intact areas of antral mucosa of human stomach [1]. The organism colonizes the non-acid-secreting mucosa of the stomach and the upper intestinal tract, including the duodenum. It survives in the acidic conditions of the stomach by generating ammonia from urea. The ammonia is believed to neutralize gastric acidity around the *H. pylori*. Person-to-person contact and ingestion of contaminated food or water are probable mechanisms for transmission of *H. pylori* [2].

Recent studies have revealed that *H. pylori* causes persistent infection in the human gastrointestinal tract and induces chronic gastritis, peptic ulcers, gastric cancer, and malignant lymphoma [3–5]. Furthermore, *H. pylori *was reported to induce vigorous humoral and cellular immune responses. White blood cell, neutrophil or lymphocyte counts were reported to increase in *H. pylori-*infected patients [6, 7]. Conflicting data were reported on the percentages of B and T cell subsets in *H. pylori-*infected patients. One study reported no significant change in the percentages of B cell, CD4, CD8, or NK cell in peripheral blood lymphocytes [8, 9]. On the other hand, Soares et al. reported increased peripheral blood CD4, activated CD4 and CD8 cells in *H. pylori-*infected patients [10].

*H. pylori* colonization of the gastric mucosa promotes severe local inflammation and induces recruitment of CD4^+^ and CD8^+^ cells [11]. Th1 cells also accumulate in the gastric mucosa of *H. pylori-*infected patients and are reported to promote mucosal inflammation by secretion of IFN-gamma [12].

Regulatory T cells (Tregs) are nonproliferative and/or anergic in response to polyclonal stimulation but can suppress the proliferation and cytokine production of both CD4^+^ and CD8^+^ T cells via a cell-cell contact-dependent mechanism [13]. Their surface marker phenotype has been established as CD4^+^CD25^+^Foxp3^+^ [14]. *H. pylori *colonizes gastric mucosa for an extended period despite the presence of a host immune system. Thus, it is hypothesized that the Tregs are involved in actively suppressing the host immune response. In fact, the number of Tregs in gastric mucosa of *H. pylori-*infected patients is significantly higher than in healthy controls [15–18].

In addition to gastrointestinal disease, *H. pylori* is associated with several autoimmune diseases, including idiopathic thrombocytopenic purpura (ITP), Sjögren syndrome, systemic sclerosis [19], Graves' disease [20], and autoimmune pancreatitis [21]. Given this association with autoimmune diseases, we hypothesized that *H. pylori* might induce systemic immunological changes. We, therefore, compared the following immune characteristics in peripheral blood of *H. pylori-*infected patients and control subjects: CD4^+^ T cells, CD8^+^ T cells, ratio of CD4/CD8 cells, Th1 cells, Th2 cells, ratio of Th1/Th2 cells, CD4^+^CD25^+^ T cells, and CD4^+^CD25^+^Foxp3^+^ T cells.

## 2. Patients and Methods

### 2.1. Patients

This study was performed with approval from the Human Research Ethical Committee of Gunma University Hospital, and all patients and volunteers gave informed consent. Forty-five *H. pylori-*infected patients (32 male, 13 female) diagnosed at Gunma University Hospital between 2007 and 2010 were included in this study. The median age of the patients was 58 years (range, 22–81 years), and the male-female ratio was 32/13. Infection by *H. pylori* was established by the following criteria: positive tests for urease and/or the biopsy sample of the stomach. Controls were obtained from 21 *H. pylori*-uninfected donors, who were negative for serum anti-*H. pylori* IgG antibody (Eiken Chemical Co., Ltd, Tokyo, Japan). The male/female ratio of controls was 1.1 (11/10) and the median age was 36 years (range, 23–65 years). The clinical data for study subjects are shown in Table 1.

### 2.2. Measurement of CD4/CD8 Ratio

Peripheral blood cells were collected using EDTA-3 K. Lymphocytes were labeled using the following monoclonal antibodies: CD45-FITC/CD4-RD1/CD8-ECD/CD3-PC5 (Cyto-Stat tetraCHROME monoclonal antibodies) and subsets were analyzed using an EPICS XL System II (Beckman Coulter, Fullerton, CA).

### 2.3. Measurement of Th1/Th2 Ratio

Whole heparinized blood was incubated at 37°C with 7% CO_2_ for 4 hours with 25 ng/mL phorbol 12-myristate 13-acetate (Sigma-Aldrich, St. Louis, MO), 1 *μ*g/mL ionomycin, and 10 *μ*g/mL Brefeldin A (Sigma-Aldrich, St. Louis, MO).

After treatment with FACS lysing solution and FACS permeabilizing solution (BD Biosciences, San Jose, CA), cells were stained at 4°C for 30 min with antihuman CD4-PE-Cy5 (BD Pharmingen, San Jose, CA), FastImmune fluorescein isothiocyanate (FITC)-labeled antihuman IFN-*γ*, and phycoerythrin- (PE-) labeled antihuman IL-4 (BD Biosciences, San Jose, CA). FastImmune IgG2a FITC/IgG1 PE isotype controls (BD Biosciences, San Jose, CA) were used as negative controls for human IFN-*γ* and IL-4. Measurement of Th subsets was performed by 3-color flow cytometry as described below for Treg cells. 

### 2.4. Treg Cell Measurements

PE-Cy5 antihuman CD4 monoclonal antibody (BD Pharmingen, San Diego, CA) and FITC antihuman CD25 (BD Biosciences, San Jose, CA) were used for surface antigen staining. Mouse IgG1, *κ*-FITC (BD Pharmingen) was used as an isotype control. PE-conjugated antihuman Foxp3 (PCH101) and PE-conjugated rat IgG2a isotype control from eBiosciences (San Diego, CA) were used for intracellular Foxp3 staining according to the manufacturer's instructions. Tregs were defined as CD4^+^CD25^+^Foxp3^+^ cells. Three-color flow cytometric analysis was performed on FACS Calibur flow cytometer using Cell Quest software (BD Biosciences, San Jose, CA).

### 2.5. Complete Blood Cell Count

Complete blood cell counts were performed using a total blood analyzer (ADVIA 120, Siemens, Tarrytown, NY).

### 2.6. Statistical Analysis

Data analysis was performed using SPSS statistics 17.0 (IBM, USA). Comparisons of quantitative data were performed using a nonparametric test (Mann-Whitney *U* test). *P* values < 0.05 were considered significant.

## 3. Results

### 3.1. Patient Populations

The clinical data for study subjects is shown in Table 1. The median age was significantly higher in *H. pylori-*infected patient group compared to the control group. However, there were no significant differences between the two groups in other items. White blood cell, neutrophil, monocyte, and lymphocyte counts were statistically identical in *H*.* pylori-*infected patients and control subjects.

### 3.2. Percentage of CD4^+^ T Cells and CD8^+^ T Cells, and CD4/8 Ratio

**Figure 1:**
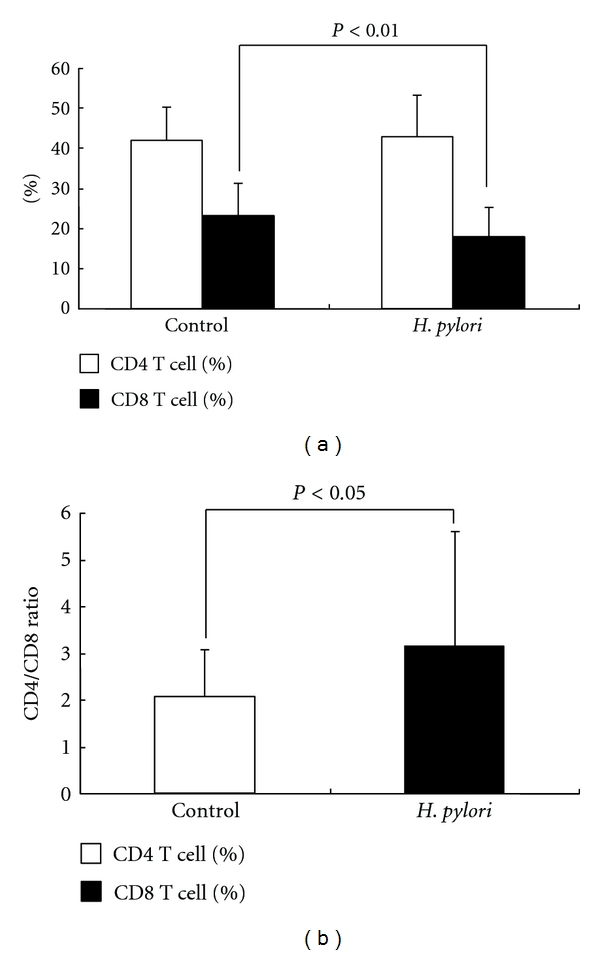
(a) Comparison of the percentage of CD4^+^ and CD8^+^ T cells. Left and right sides show the data of control subjects and *H. pylori-*infected patients, respectively. White columns represent the percentage of CD4^+^ T lymphocyte, and black columns represent CD8^+^ T lymphocyte. Data are given as mean ± SD. There was no significant difference in the percentage of CD4^+^ T cells in *H. pylori*-infected patients (mean ± SD; 43.0 ± 10.4%) as compared to control subjects (mean ± SD; 42.2 ± 8.1%). However, the percentage of CD8^+^ T cells was significantly lower in *H. pylori-*infected patients (mean ± SD; 18.0 ± 7.1%) compared to control subjects (mean ± SD; 23.2 ± 7.8%) (*P* < 0.05). (b) CD4/CD8 ratio in control subjects (white column) and *H. pylori-*infected patients (black column). Data are given as mean ± SD. The CD4/CD8 ratio was significantly higher in *H. pylori*-infected patients (mean ± SD; 3.1 ± 2.4) compared to control subjects (mean ± SD; 2.1 ± 1.0) (*P* < 0.05).

Peripheral blood cells were labeled with CD4 and CD8 tags and analyzed by FACS. We found no significant difference in the percentage of CD4^+^ T cells in *H. pylori*-infected patients (mean ± SD; 43.0 ± 10.4%) as compared to control subjects (mean ± SD; 42.2 ± 8.1%). However, the percentage of CD8^+^ T cells was significantly lower in *H. pylori-*infected patients (mean ± SD; 18.0 ± 7.1%) compared to control subjects (mean ± SD; 23.2 ± 7.8%) (*P* < 0.05) (Figure 1(a)).

Reflecting the decrease in percentage of CD8^+^ cells, the CD4/CD8 ratio was significantly higher in *H. pylori*-infected patients (mean ± SD; 3.1 ± 2.4) compared to control subjects (mean ± SD; 2.1 ± 1.0) (*P* < 0.05) (Figure 1(b)).

### 3.3. Percentage of Th1 and Th2 Cells, and Th1/Th2 Ratio

**Figure 2:**
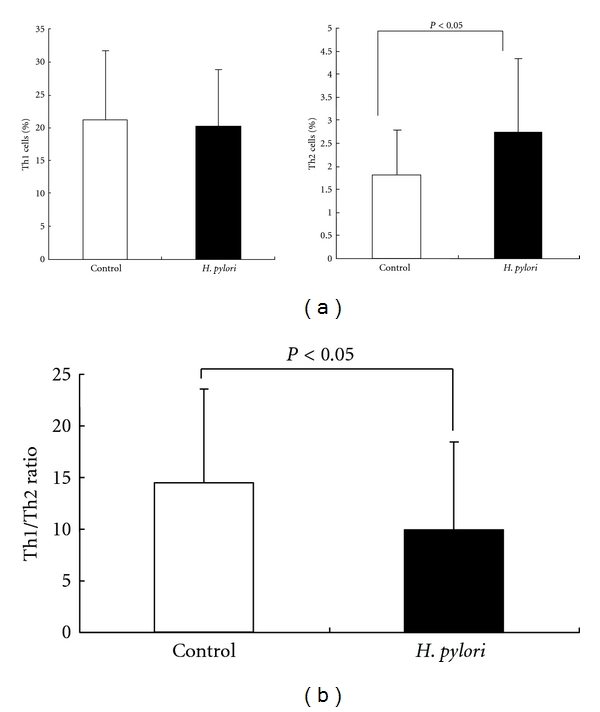
(a) The percentage of Th1 cells and Th2 cells. Left and right figures show the percentage of Th1 and Th2 cells, respectively. White columns represent the data of normal subjects, and black columns represent the data of *H. pylori-*infected patients. Data are given as mean ± SD. There was no significant difference in the percentage of Th1 cells in *H. pylori-*infected patients (mean ± SD; 20.2 ± 8.6%) and control subjects (mean ± SD; 21.2 ± 10.6%). However, the percentage of Th2 cells in *H. pylori*-infected patients (mean ± SD; 2.8 ± 1.6%) was significantly higher than in control subjects (mean ± SD; 1.8 ± 1.0%), (*P* < 0.05). (b) Comparison of Th1/Th2 ratio. White columns represent the data of normal subjects, and black columns represent the data of *H. pylori-*infected patients. Data are given as mean ± SD. The Th1/Th2 ratio was significantly lower in *H. pylori*-infected patients (mean ± SD; 10.0 ± 8.5) compared to control subjects (mean ± SD; 14.5 ± 9.0) (*P* < 0.05).

Peripheral blood cells were stained for FACS analysis with markers for CD4 as well as intracellular stains for IFN-*γ* and IL-4 to identify Th1 and Th2 cells, respectively. There was no significant difference in the percentage of Th1 cells in *H. pylori-*infected patients (mean ± SD; 20.2 ± 8.6%) and control subjects (mean ± SD; 21.2 ± 10.6%). However, the percentage of Th2 cells in *H. pylori*-infected patients (mean ± SD; 2.8 ± 1.6%) was significantly higher than in control subjects (mean ± SD; 1.8 ± 1.0%), (*P* < 0.05) (Figure 2(a)). Consistent with the increase in Th2 cells, the Th1/Th2 ratio was significantly lower in *H. pylori*-infected patients (mean ± SD; 10.0 ± 8.5) compared to control subjects (mean ± SD; 14.5 ± 9.0) (*P* < 0.05) (Figure 2(b)).

### 3.4. Percentage CD4^+^CD25^+^ and CD4^+^CD25^+^Foxp3^+^ T Cells

**Figure 3:**
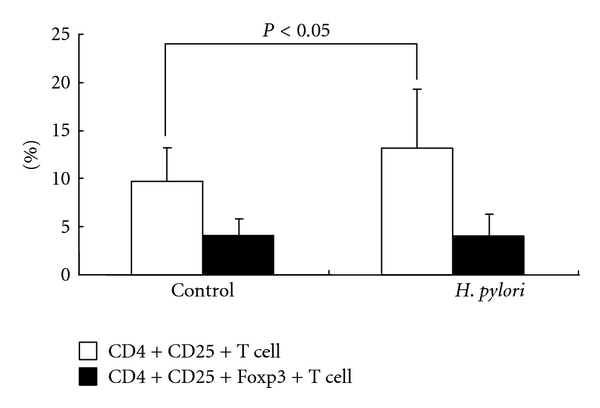
The percentage of CD4^+^CD25^+^ T cell and CD4^+^CD25^+^Foxp3^+^ (regulatory T cell). Left and right sides show the data of control subjects and *H. pylori-*infected patients, respectively. White columns represent the percentage of CD4^+^CD25^+^ T cell, and black columns represent the percentage of regulatory T cell. Data are given as mean ± SD. The percentage of CD4^+^CD25^+^ T cells in *H. pylori-*infected patients (mean ± SD; 13.2 ± 6.2%) was significantly higher than that in control subjects (mean ± SD; 9.8 ± 3.4%) (*P* < 0.05). The percentage of Tregs (CD4^+^CD25^+^Foxp3^+^ T cells) was not significantly different between *H. pylori*-infected patients (mean ± SD; 4.1 ± 2.1%) and control subjects (mean ± SD; 4.0 ± 1.7%).

The percentage of CD4^+^CD25^+^ T cells in *H. pylori-*infected patients (mean ± SD; 13.2 ± 6.2%) was significantly higher than that in control subjects (mean ± SD; 9.8 ± 3.4%) (*P* < 0.05) (Figure 3). This cell population includes both activated CD4^+^ T cells and Tregs and thus changes in Tregs could be masked in this impure population. To measure the percentages of the Tregs, we monitored the frequency of CD4^+^CD25^+^Foxp3^+^ T cells. The percentage of these Tregs was not significantly different between *H. pylori*-infected patients (mean ± SD; 4.1 ± 2.1%) and control subjects (mean ± SD; 4.0 ± 1.7%) (Figure 3).

### 3.5. The Difference of T Lymphocyte Subsets between Young and Old Subjects in the Control Group

Because the mean age of the control subjects was lower than that of the patient group, the control subjects were divided into 2 groups: <40 years old (*n* = 13) and ≥40 years old (*n* = 8). When comparing the mean percentage of T lymphocyte subsets, there were no significant differences between the <40 group and the ≥40 group (Table 2). 

## 4. Discussion

Infection of the gastric mucosa with *H. pylori* is thought to occur in early childhood and to play a key role in the pathogenesis of many gastroduodenal diseases, including gastric ulcers, duodenal ulcers, gastric cancer, and gastric lymphoma. Previous studies have shown that *H. pylori* infection induces vigorous humoral and cellular immune responses. Kondo et al. reported that patients with *H. pylori *infection had increased neutrophile and monocyte counts in their peripheral blood [6]. Karttunen et al. also reported that white blood cell and lymphocyte counts were increased in *H. pylori-*infected patients [7]. In contrast, in our report, we found no significant difference in white blood cell, neutrophil, lymphocyte, and monocyte counts between *H. pylori*-infected patients and control subjects. Because *H. pylori*-infection induces chronic and local inflammation in gastric mucosa, it might not influence the absolute blood cell counts.

 Yuceyar et al. reported no significant difference between *H. pylori-*infected patients and normal subjects in the peripheral lymphocyte profile, including T-, B-, CD4^+^ T-, CD8^+^ T- and NK cells [9]. Conversely, the data presented here, show a decrease in the percentage of CD8^+^ T cells and an increase in the CD4/CD8 ratio in peripheral blood cells of *H. pylori*-infected patients. CD8^+^ T cells have also been reported to accumulate in the gastric mucosa and participate in the inflammatory process, promoting the severity of the disease [22, 23]. These studies corroborate the findings of our study which suggests that the distribution of CD8^+^ T cells changes in *H. pylori*-infected patients, with peripheral CD8^+^ T cells migrating into the gastric mucosa.

 It is well known that* H. pylori* induces T-cell polarization towards a Th1-dominant response in gastric mucosa [24]. Moreover, D'Elios showed that *H. pylori*-specific Th1 cells could be cloned from *H. pylori-*infected gastric mucosa and that these cells were cytotoxic to gastric epithelial cells as they produced IFN-*γ* [12]. However, few reports have been published to date characterizing the Th1/Th2 ratio in peripheral blood of *H. pylori-*infected patients. We find that the Th1/Th2 ratio in peripheral blood is significantly decreased in *H. pylori-*infected patients as compared to normal subjects. We hypothesize that, as suggested for the CD8^+^ T cells, peripheral blood Th1 cells migrate to the gastric mucosa in *H. pylori-*infected patients.

Age-related difference in Tregs has been described controversially [25]. Rosenkranz et al. reported that the frequency of Tregs (CD4^+^Foxp3^+^) increased with aging [26], and Gregg et al. also reported an increase in peripheral blood CD4^+^CD25 high regulatory T cells associated with aging [27]. Conversely, others reported no correlation between the number of circulating CD4^+^CD25 high Tregs and age [28]. In our study, we found that there was no statistical difference in the mean number of Tregs between <40-year-old and >40-year-old control subjects. Therefore, it was considered that the difference of age between control group and the patient group did not affect the evaluation of Tregs in *H. pylori-*infected patients.

There was no report about the number/ratio of peripheral CD4^+^CD25^+^Foxp3^+^ Tregs in *H. pylori*-infected patients. Lundgren et al. reported that *H. pylori-*infected patients have an impaired memory CD4^+^ T cell response to *H. pylori* that is linked to the presence of *H. pylori*-specific CD4^+^25^+^ T cells in peripheral blood, which actively suppress the response [29]. These authors also demonstrated the presence of Tregs that expressed the characteristic CD4^+^25^+^ surface markers and coexpressed high levels of Foxp3 mRNA in the gastric mucosa [15]. In the data reported here the percentage of peripheral blood CD4^+^CD25^+^ T cells, but not CD4^+^25^+^Foxp3^+^ T cells, increased in *H. pylori-*infected patients. Since CD4^+^CD25^+^ T cells include Tregs as well as activated T cells, our data indicate that the percentage of Tregs did not increase in the peripheral blood of *H. pylori-*infected patients.

 Tregs did not decrease, but the activation of humoral immunity and Th2 polarization were observed in the peripheral blood of *H. pylori-*infected patients. In some cases, these changes may induce systemic autoimmune diseases.

## Figures and Tables

**Table 1:** Characteristics of participants in this study.

	Control	*H. Pylori*
Cases (*n*)	21	45
Sex (male: female)	(9 : 9)	(32 : 13)
Age median: range (year)	36: 23–65*	58.0: 22–81*
Hemoglobin (g/dl)	13.7 ± 1.2	13.8 ± 1.6
Hematocrit (%)	41.4 ± 4.0	41.0 ± 3.5
WBC (×10^9^/l)	5.7 ± 1.5	6.3 ± 1.8
Neutrophils (×10^9^/l)	3.2 ± 1.2	3.8 ± 1.4
Monocytes (×10^9^/l)	0.32 ± 1.1	0.35 ± 1.1
Lymphocytes (×10^9^/l)	1.8 ± 0.5	1.8 ± 0.5
Platelets (×10^9^/l)	233.9 ± 55.4	241.0 ± 67.9

mean ± SD *P values < 0.05.

**Table 2:** The difference of T-lymphocyte subsets between young and old subjects in control group.

	Age < 40	Age ≥ 40	*P* values
Cases (*n*)	13	8	
CD4 T cells (%)	43.2 ± 7.3	40.7 ± 9.4	0.89
CD8 T cells (%)	22.4 ± 3.9	24.4 ± 12.1	0.59
CD4/CD8 ratio	2.0 ± 0.4	2.3 ± 1.6	0.89
Th1 cells (%)	19.1 ± 10.1	24.6 ± 11.3	0.096
Th2 cells (%)	1.7 ± 0.8	2.0 ± 1.2	0.66
Th1/Th2 ratio	14.0 ± 9.0	15.2 ± 9.6	0.83
CD4^+^CD25^+^ T cells (%)	9.7 ± 3.6	10.0 ± 3.4	0.66
CD4^+^CD25^+^Foxp3^+^ T cells (%)	3.9 ± 1.8	4.3 ± 1.5	0.66
